# The Mediating Role of Romantic Desolation and Dating Anxiety in the Association Between Interpersonal Competence and Life Satisfaction Among Polish Young Adults

**DOI:** 10.1007/s10804-015-9216-3

**Published:** 2015-09-15

**Authors:** Katarzyna Adamczyk, Chris Segrin

**Affiliations:** Institute of Psychology, Adam Mickiewicz University, ul. A. Szamarzewskiego 89/AB, 60-568 Poznan, Poland; Department of Communication, University of Arizona, Tucson, AZ 85721 USA

**Keywords:** Romantic desolation, Relationship status, Romantic loneliness, Dating anxiety, Life satisfaction, Interpersonal competence

## Abstract

This study investigates the role of romantic desolation on life satisfaction in young adulthood. Using data from a Polish sample of 330 (205 females and 125 males) young adults aged 20–30, who completed Polish versions of the Satisfaction With Life Scale, Dating Anxiety Scale, Interpersonal Competence Questionnaire-Revised, and Social and Emotional Loneliness Scale for Adults-Short Form, romantic desolation (romantic loneliness and lack of a romantic partner) and dating anxiety were tested as mediators of the association between interpersonal competence and life satisfaction. Results revealed that single individuals reported lower life satisfaction and higher romantic loneliness than did partnered individuals. At the same time, no differences emerged between single and partnered individuals in dating anxiety or interpersonal competence. Structural equation modeling results showed that low interpersonal competence has an indirect effect on romantic desolation through higher levels of dating anxiety. Also, dating anxiety had an indirect effect on lower life satisfaction through increased romantic desolation. These results highlight the important role of dating anxiety and romantic desolation for explaining why low interpersonal competence is associated with diminished life satisfaction in young adults.

## Introduction

Romantic relationships are one of the most important facets of people’s social environment (Lehnart et al. 2010). The formation of intimacy and dating relationships has high importance in young adulthood (Beisert 1991; Masten et al. 2004), when individuals typically form enduring romantic relationships (Donnellan et al. 2005). Therefore, romantic relationships are recognized to be an important factor for emotional well-being in early adulthood (Simon and Barret 2010). Moreover, happiness (or life satisfaction) in young adulthood may be dependent on the successful achievement of developmental tasks specific to this stage of the lifespan such as establishment of marital or other long term intimate relationships (Havighurst 1981; Martikainen 2009).

Establishing intimacy in romantic relationships is one of the fundamental developmental tasks in young adulthood (Conger et al. 2000; Havighurst 1981). Competence in intimate relationships is also a vital indicator of healthy psychosocial functioning (Larson et al. 2007). Larson et al. (2007) found that young adults’ social competence was associated with such indices of psychosocial functioning as higher self-esteem, educational attainment, and ego development, and lower psychological symptoms and criminal behavior. Given the rise in singlehood in Europe, including Poland, and the US (Poortman and Liefbroer 2010; Such-Pyrgiel 2014) and the role of romantic relationships in predicting personal well-being (Conger et al. 2000), it is important to gain a better understanding of potential factors that may be associated with romantic involvement and individual well-being in young adulthood. At the same time, there is also a need to examine the functions and correlates of social skills in more diverse populations, especially among those in differing life circumstances (Segrin and Taylor 2007) such as people seeking to form romantic attachments. Accordingly, a major aim of this investigation is to explore the role of interpersonal competence in facilitating establishment of these relationships and their consequences for life satisfaction in young adults.

### Interpersonal Competence

In young adulthood social interactions and formation of romantic relationships require a range of interpersonal competencies (e.g., Grover et al. 2005; Klaus et al. 1977). The inability to form and maintain satisfying relationships may lead to a sense of loneliness (Kuczyńska and Dolińska-Zygmunt 2004). For example, students who do not date at all are more lonely and perceive themselves to have lesser communication skills than those students who regularly date others (Prisbell 1988). Studies conducted in Poland also reveal that people with romantic partners generally have higher interpersonal competence than those who do not have a partner (e.g., Adamczyk and Pilarska 2012; Górska 2011).

Interpersonal competence is associated with greater life satisfaction, environmental mastery, self-efficacy in social situations, hope, happiness, and quality of life (Segrin et al. 2007; Segrin and Taylor 2007). Moreover, the successful realization of developmental tasks in young adulthood, especially in the domain of close relationships, requires a range of interpersonal skills (Brzezińska 2008; Steca et al. 2009). People with good interpersonal competence have greater chances to develop satisfying marital relations and to build a network of social support (Armistead et al. 1995; Buhrmester et al. 1988). As a result, it is reasonable to expect that young adults possessing high level of interpersonal competence will be more likely to successfully manage their dating activity and romantic relationships than young adults who display a lower level of interpersonal competence (see Curran et al. 1976). As a consequence, a high level of interpersonal competence that facilitates success in dating and romantic domains should be related to higher life satisfaction.

### Dating Anxiety

During young adulthood people often seek a partner with whom they will spend a substantial part of later life (Meeus et al. 2007) and one means of doing so is dating (Allen et al. 1998). Unfortunately, for some young adults, dating is associated with inherent anxiety that sometimes results in avoidance (Allen et al. 1998). Dating anxiety is a significant problem among college students and adults, and feelings of anxiety and distress in dating situations can interfere with the ability to form and sustain close and intimate romantic relationships (Chorney and Morris 2008). Therefore, the inability to comfortably participate in romantic interactions may eventually lead to the development of dysfunctional patterns of behavior (Allen et al. 1998). It turn, the anxiety experienced in dating situations may be to some degree the result of negative consequences anticipated by an individual, which are caused by deficits in the social skills necessary for successful dating interactions (Curran et al. 1976). Consequently, dating anxiety may prevent many young adults from establishing romantic partnerships (La Greca and Mackey 2007).

### Romantic Involvement

Formation of a deep bond with a partner constitutes a benchmark of social and emotional maturity in young adulthood (Seiffge-Kranke 2003). Social roles associated with marriage, parenting, and work life define the place of an individual in the world of adults (Matuszewska 2003), and contribute to achievement of higher personality maturity, as well as intellectual and social-moral maturity (Lehnart et al. 2010; Rostowska 2008). The lack of romantic partners or intimate relationships may be an important causal factor for feelings of loneliness (e.g., Rokach and Brock 1998). Loneliness is often conceptualized as a multifaceted and domain-specific phenomenon. Weiss (1973) was the first to describe loneliness as a multidimensional experience and proposed a distinction between *social loneliness* resulting from inadequate access to social relationships such as a network of peers, co-workers, neighbours, or friends, and *emotional loneliness* resulting from lack of close or intimate relationships as one might experience with a romantic partner, parent, or child. Emotional loneliness is primarily related to “the absence of a partner, that is, with the absence of an exclusive, close, and intimate tie” (Dykstra and Fokkema 2007, p. 9). In turn, social loneliness is related to a perceived deficiency in social networks, or a lack of social relations or social activities (Russell et al. 1984; Weiss 1973). Furthermore, on the basis of Weiss’ (1973) distinction between the experience of social isolation (social loneliness) and emotional isolation (emotional loneliness), DiTommaso and Spinner (1993) noted that emotional loneliness appeared to be comprised of two domains, that is, family emotional loneliness and romantic emotional loneliness. As prior studies have shown, married individuals and individuals living with a significant other reported less romantic loneliness than those who were not in such relationships (Bernardon et al. 2011). Also, being involved in a romantic relationship was significantly related to lower levels of romantic loneliness, but was only weakly linked to family and social loneliness (DiTommaso and Spinner 1993).

Establishment of intimacy in romantic relationships is one of the fundamental developmental tasks in young adulthood (Conger et al. 2000; Havighurst 1981) and lack of involvement in romantic relationships is associated with romantic loneliness. Thus, it stands to reason that establishment of marriage or other long term intimate relationships (i.e., romantic involvement) has been found to be positively associated with happiness (or life satisfaction) in young adulthood (e.g., Martikainen 2009). Therefore, it is plausible to assume that the lack of a romantic partner and a high level of romantic loneliness can contribute to lower life satisfaction.

### Life Satisfaction

Life satisfaction is a major indicator of quality of life (Veenhoven 1996). It is one of the components of subjective well-being that refers to a cognitive judgment beyond mere positive and negative affect (Diener et al. 1985). In addition, this judgment of satisfaction is based on comparison with standards set by an individual that are not imposed by others (Diener et al. 1985). A vast body of research shows that romantic relationship involvement is associated with greater well-being among adults (Umberson and Williams 1999). For example, married individuals report higher levels of well-being than do individuals who never married, or were separated, divorced, or widowed (Kamp Dush and Amato 2005). Moreover, as Veenhoven (1996) indicated, in all modern societies single individuals report less pleasure with life than married individuals, and this difference in the extent of life satisfaction between single and partnered persons is greater than that expressed between rich and poor.

Therefore, if the basis of this cognitive judgment is one’s own individual judgment but not an external criterion (Diener 1984), it is reasonable to assume that a substantial basis for life satisfaction in young adulthood is romantic relationship involvement. This is because lasting, successful relationships are often central to people’s values (Adler et al. 2006; Engels et al. 2001), and because the achievement of intimacy in romantic relationships constitutes one of the fundamental developmental tasks of young adulthood (Conger et al. 2000; Havighurst 1981). Happiness (or life satisfaction) in young adulthood may be dependent on the successful achievement developmental tasks specific to this stage of the lifespan such as establishment of marriage or other long term intimate relationships (Martikainen 2009). Indeed, Martikainen (2009) and also Kuusinen’s (1997) study confirmed that life satisfaction of the young adults was related to success in developmental tasks distinguished by Havighurst (1981) such as establishing intimate relationships and starting a working career.

## The Current Study

To the best of our knowledge, the current study is the first to test dating anxiety and romantic involvement as mediators of the association between interpersonal competence and the life satisfaction of young adults. This study extends previous research in several ways. First, by testing potential mediators of the association between interpersonal competence and life satisfaction, it provides a potential explanation for why people with high levels of interpersonal competence often experience high life satisfaction. Second, it addresses a critical need for research on life satisfaction in earlier developmental stages of the lifespan (Steca et al. 2009). Rather than comparing single to married people, as is common in the literature, we compared single individuals with individuals in nonmarital intimate relationships as these relationships play a crucial role in young adults’ lives, identity, self-concept, and psychological well-being (Simon and Barret 2010). Third, follwing by the call for the assessment of dating anxiety in diverse populations (Chorney and Morris 2008; Grover 2008), we assessed dating anxiety which is recognized to be a precursor to numerous psychological difficulties in the yound adult population. Fourth, in the current investigation we employed the multidimensional approach to assessing interpersonal competence and dating anxiety in order to capture multiple elements of these experiences and their associations with life satisfaction and romantic involvement. In addition, we focused on a particular type of emotional loneliness, that is romantic loneliness, which results from lack of a romantic partner. Finally, the vast majority of studies concerning civil status and its outcomes come from the USA. Consequently, there is a need to explore and replicate these associations in other cultures to expand the scope and generalizability of the findings. Accordingly, the following predictions will be tested in a Polish sample of young adults:

### **H1**

Young adults in committed romantic relationships will exhibit higher life satisfaction than their single peers.

### **H2**

Young adults in committed romantic relationships will report lower level of romantic loneliness than those who are single.

### **H3**

Young adults in committed romantic relationships will report lower level of dating anxiety than single peers.

### **H4**

Young adults in committed romantic relationships will exhibit higher level of interpersonal competence than those who are single.

In addition, we also tested an omnibus theoretical model that began by specifying a negative association between interpersonal competence and dating anxiety. One of the many benefits of interpersonal competence is comfort in social situations, and this is expected to be manifest in lower dating anxiety. Dating anxiety was, in turn, assumed to be associated with a latent variable that we characterize as “romantic desolation.” This is an amalgamation of romantic loneliness and lack of a romantic partner. Finally, both dating anxiety and romantic desolation were assumed to have negative effects on life satisfaction. A key element of this structural model is the ability to test two mediators, or indirect effects, on life satisfaction. Specifically, we predict that:

### **H5**

Interpersonal competence will have an indirect effect on life satisfaction, through lower dating anxiety.

### **H6**

Dating anxiety will have an indirect effect on life satisfaction through romantic desolation.

In this model, romantic involvement and dating anxiety provide a theoretical link between possession of interpersonal competence and life satisfaction. According to Havighurst’s (1981) developmental tasks theory, and Martikainen (2009) research, life satisfaction of young adults is related to success in developmental task achievement, including establishing intimate relationships. Successful achievement of this developmental task requires possessing of social skills to form and to sustain intimate and social relationships (Steca et al. 2009). Deficits in the interpersonal competencies may fuel anxiety experienced in dating situations (Curran et al. 1976) and of course this anxiety and distress in dating situations may interfere with the ability to form and sustain close and intimate romantic relationships (Chorney and Morris 2008), and promote feelings of loneliness (Rokach and Brock 1998), both of which are potentially deleterious to life satisfaction.

## Method

### Participants and Procedure

The study was conducted on a sample of 188 university students from different faculties at Adam Mickiewicz University in Poznan, Poland (57 %) and 142 non student participants (43 %) who resided in a large Polish city with a population exceeding 500,000. The age of participants ranged from 20 to 30 years old, with the average age of 22.64 (*SD* = 3.18). All the respondents were heterosexual, unmarried, and had no children. Women represented 62.12 % (*n* = 205) of the sample and men represented 37.88 % (*n* = 125). One hundred ninety-one participants (57.90 %) reported being in a romantic relationship at the time of the assessment, while 139 participants (42.10 %) were not.

To recruit participants, the first author distributed questionnaires through university students who were also asked to refer members of their social networks to participate in the investigation. The questionnaire packages were administered in classrooms to groups of 20–30 students at a time and participation was voluntary. The nonstudent participants were obtained through university students who passed questionnaires to members of their social networks. At the same time, university students were specifically instructed to not recruit their romantic partners into the study, but they were allowed to recruit friends or other young adult family members. The purpose of the study was explained to participants along with an assurance of anonymity and confidentiality and explanation of their freedom to withdraw from the study without consequence. The study was conducted according to the ethical guidelines in the Polish Code of Professional Ethics for the Psychologist that apply to psychologists who are researchers and practitioners.

### Measures

Measures for this investigation were selected based on their representation of the constructs specified in the hypotheses (e.g., life satisfaction, romantic loneliness), a record of psychometric quality (e.g., reliability, validity), and prior translation and use in the Polish language. The questionnaire presented to participants was comprised of the following instruments.

*Demographic Questionnaire* A series of demographic questions was asked to obtain general descriptive information about participants’ background such as their age, sex, education level, and current relationship status. To determine the current relationship status, participants were asked to answer “yes” or “no” to the question whether they have a romantic partner.

*Dating Anxiety Scale for Adolescents* (DAS-A; Glickman and La Greca 2004) (Polish adaptation for adults—Adamczyk 2015). The original DAS-A assesses adolescents’ anxiety in heterosocial and dating situations. It contains of 21 items rated on a five-point scale ranging from 1 (*not at all characteristic of me*) to 5 (*extremely characteristic of me*) with additional five filler items. The questionnaire is comprised of the following three subscales: Fear of negative evaluation–dating (FNE–Dating; concern or worry that a date or a member of the opposite sex would judge the self in a negative manner), social distress–dating (SD–Date; distress while interacting with a member of the opposite sex on a date or social occasion), and social distress–group (SD–Group; inhibition and distress during heterosocial group situations). Glickman and La Greca (2004) investigated and found the subscales to have good internal consistency: .94 for total DAS-A, .92 for FNE–Dating, .88 for SD–Date, and .81 for SD–Group. The Cronbach’s alphas in the present study were .92 for FNE–Dating, .89 for SD–Date, .81 for SD–Group, and .95 for the total DAS.

*Interpersonal Competence Questionnaire-Revised* (ICQ-R; Buhrmester et al. 1988) (Polish adaptation—Górska 2011). The ICQ-R is a measure of interpersonal competences in specific social situations, mostly relevant to intimate relationships. The ICQ-R is a measure of interpersonal competences in specific social situations, mainly direct, informal and intimate relationships. It consists of 40 items rated on a five-point Likert-type scale, ranging from 1 (*strongly disagree*) to 5 (*strongly agree*). This questionnaire is comprised by the five following subscales: Initiating relationships, Asserting influence, Self-disclosure, Providing emotional support, and Conflict resolution. The score for each scale is the average scale score. The above-mentioned dimensions were found to be independent and have satisfactory psychometric parameters with internal reliability estimates from .77 to .87 (Buhrmester et al. 1988). The Polish language version by Górska (2011) has been shown to be a valid measure of interpersonal competence in a Polish population with Cronbach’s alphas ranging from .72 to .86. The Cronbach’s alphas in the present study were .86 for Initiating relationships, .85 for Providing emotional support, .84 for Asserting influence, .86 for Self-disclosure, and .76 for Conflict resolution.

*Satisfaction with Life Scale* (SWLS; Diener et al. 1985) (Polish adaptation—Juczyński 2009). This scale measures an individual’s satisfaction with his/her life. The SWLS uses a seven-point Likert scale, ranging from *strongly disagree* (1) to *strongly agree* (7). The scale’s internal consistency is high (α = .87) and 2-week test–retest reliability was *r* = .85 (Diener et al. 1985). The Cronbach’s alpha in the current study was .83.

*The Social and Emotional Loneliness Scale for Adults*-*Short Form* (SELSA-S; DiTommaso et al. 2004) (Polish adaptation—Adamczyk and DiTommaso 2014). The SELSA-S is a multidimensional measure of loneliness which consists of 15 items rated on a seven-point Likert-type scale, ranging from 1 (*strongly disagree*) to 7 (*strongly agree*). It was designed to measure emotional (romantic and family) and social loneliness within the past year. The family loneliness subscale assesses feelings toward family relationships. The social loneliness subscale measures feelings toward being part of a social group. The romantic loneliness subscale measures the degree to which participants feel they have significant others in their lives. The three subscales of the SELSA-S have high internal reliability, with Cronbach’s alpha coefficients ranging from .87 to .90, and have been shown to be valid measures of loneliness (DiTommaso et al. 2004). In the current study we used the subscale measuring romantic loneliness and its Cronbach’s alpha was .83.

## Results

### Mean-Level Analyses

An analysis of variance and one-way MANOVA were used to examine mean differences between single and partnered individuals on life satisfaction, romantic loneliness, dating anxiety, and interpersonal competence. Table 1 displays the means and standard deviations of the variables.

**Table 1:** Means and SDs on interpersonal competence, dating anxiety, romantic loneliness, and life satisfaction by relationship status

Variables	Total sample (N = 330) Mean (SD)	Single individuals (*n* = 139) Mean (SD)	Partnered individuals (*n* = 191) Mean (SD)	*F* value	Effect size
Life satisfaction	21.97 (5.35)	21.22 (5.57)	22.52 (5.13)	4.82*	.24
Romantic loneliness	14.69 (7.94)	21.10 (6.07)	10.03 (5.53)	296.77***	1.91
Multivariate test^†^				2.32	.02^†^
*Dating anxiety*					
Fear of negative evaluation–dating	29.12 (9.10)	30.17 (9.48)	28.36 (8.77)	3.18	.01
Social distress–dating	19.69 (6.20)	20.45 (6.51)	19.14 (5.92)	3.66	.01
Social distress–group	9.70 (3.77)	9.68 (3.71)	9.71 (3.83)	.01	.00
Total dating anxiety	58.51 (17.32)	60.30 (17.89)	57.21 (16.81)	2.58	.01
Multivariate test^†^				1.88	.03^†^
*Interpersonal competence*					
Initiating Relationships	24.85 (5.04)	24.42 (5.27)	25.16 (4.86)	1.75	.01
Providing emotional support	26.24 (4.44)	26.14 (4.86)	26.32 (4.12)	.13	.00
Asserting influence	25.90 (4.76)	25.91 (4.93)	25.90 (4.65)	.00	.00
Self-disclosure	20.98 (5.65)	20.26 (5.81)	21.51 (5.49)	3.95*	.01
Conflict resolution	23.61 (4.14)	23.79 (4.33)	23.48 (4.00)	.46	.00

Effect sizes are Cohen’s *d* unless indicated by ^†^ in which case they are *η* ^2^

*** *p* < .001; * *p* < .05

First, an analysis of variance was conducted to examine whether single and partnered individuals differed in regard to life satisfaction. Participants in committed relationships had a higher level of life satisfaction than single participants, *F*(1, 328) = 4.82, *p* = .029, *d* = .24, confirming H1.

Second, we examined the hypothesis that individuals in committed relationships would report lower level of romantic loneliness (H2). An analysis of variance indicated that individuals in committed relationships reported significantly less romantic loneliness than single participants, *F*(1, 328) = 296.77, *p* = .000, *d* = 1.91.

Third, a one-way multivariate analysis of variance was used to examine whether single and partnered individuals differed in regard to dating anxiety (H3). Using Wilks’s lambda as the criterion, we did not observe a significant main effect of relationship status for dating anxiety, Wilks’s Λ = .98, *F*(3, 326) = 2.32, *p* = .075, *η*^2^ = .02. There was no significant difference between the groups on any of the dating anxiety subscales: fear of negative evaluation–dating, social distress–dating, and social distress–group.

Fourth, we examined the hypothesis that individuals in committed relationships would report higher interpersonal competence than participants who were not presently in a relationship (H4). The one-way multivariate analysis treating each interpersonal competence factor as a dependent variable, did not reveal a significant multivariate effect of relationship involvement on interpersonal competence, Wilks’s Λ = .97, *F*(5, 324) = 1.88, *p* = .05, *η*^2^ = .03. No significant differences emerged between the groups on any of the five domains of interpersonal competence, that is initiating relationships, providing emotional support, asserting influence, self-disclosure, and conflict resolution.

### Mediational Analysis

**Fig. 1:**
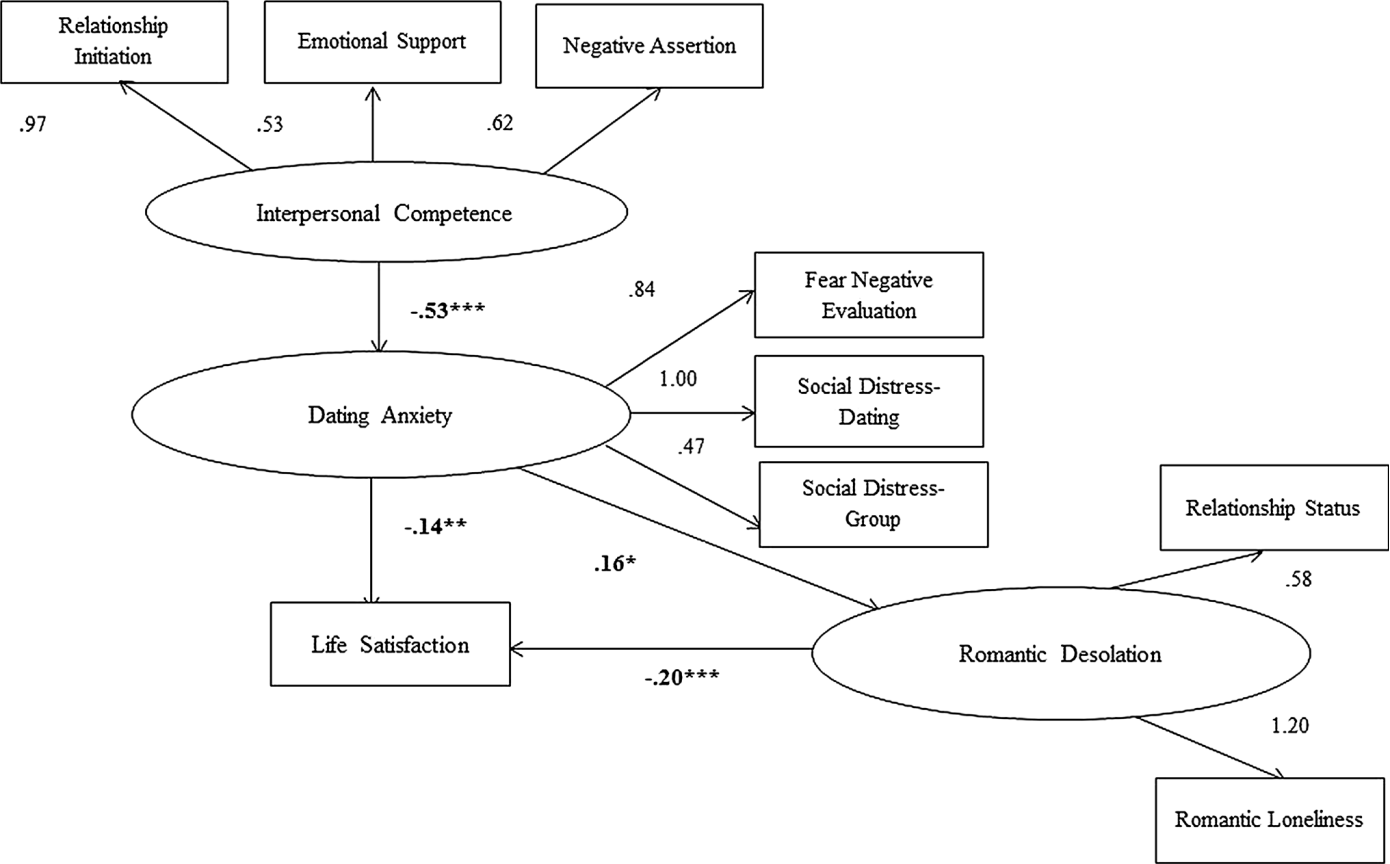
Structural model of interpersonal competence, dating anxiety, romantic desolation, and life satisfication. *Note* For ease of presentation, error terms have omitted from the model. *p < .05; **p < .01; ***p < .001

To test whether dating anxiety and relationship desolation mediate the association between interpersonal competence and life satisfaction (H5, H6), a model was testing in structural equation modeling using IBM SPSS AMOS 20.0 with maximum likelihood estimation. Before testing the structural model to assess the associations between interpersonal competence, dating anxiety, romantic desolation, and life satisfaction, we fit a measurement model that specified all possible pairwise correlations between each of these variables. Indicators of the latent variables are depicted in Fig. 1.

Our initial analysis of this measurement model indicated that the fit could be improved substantially by specifying a single correlation between the error term of the social distress–group observed variable and the interpersonal competence latent variable. With this one added specification, the resultant measurement model provided a good fit to the sample data, χ^2^ = 25.78, *df* = 20, *p* = .17, χ^2^/*df* ratio = 1.29, NFI = .98, CFI = .99, RMSEA = .03, 90 % CI .00–.06.

Next, we tested the structural model that appears in Fig. 1. Results of this analysis showed that the structural model provided a good fit to the sample data, χ^2^ = 56.34, *df* = 22, *p* ≤ .001, χ^2^/*df* ratio = 2.56, NFI = .96, CFI = .97, RMSEA = .07, 90 % CI .05–.09. As predicted, there was a significant and negative path from interpersonal competence to dating anxiety (β = −.53, *p* < .001) and from dating anxiety to life satisfaction (β = −.14, *p* < .01). Also, there was a positive association between dating anxiety and romantic desolation (β = .16, *p* < .05). Finally, there was a significant and negative direct path from romantic desolation to life satisfaction (β = −.20, *p* < .001).

Indirect effects in this structural model were estimated in AMOS 20 with a bias corrected bootstrapping procedure based on 2000 bootstrap samples. Results indicated that there was a statistically significant indirect effect of interpersonal competence on romantic desolation, through dating anxiety (β = .09, *p* < .01). There was also a statistically significant indirect effect of dating anxiety on life satisfaction through romantic desolation (β = −.03, *p* < .01).

## Discussion

This study tested predictions that individuals in committed romantic relationships would exhibit higher life satisfaction (H1), lower romantic loneliness (H2), lower dating anxiety (H3), and higher levels of interpersonal competence (H4). In addition, we tested the prediction that dating anxiety would mediate the association between interpersonal competence and life satisfaction (H5), and that romantic desolation would mediate the association between dating anxiety and life satisfaction in young adults (H6). All of the hypotheses were supported aside from H3 and H4.

Consistent with our first hypothesis, individuals in committed romantic relationships reported higher life satisfaction than single individuals. These results are congruent with prior research indicating that single individuals report less pleasure with life than married individuals (Veenhoven 1996). Moreover, this pattern of results provides support for prior research (i.e., Martikainen 2009) indicating that happiness (or life satisfaction) in young adulthood may depend on the successful achievement developmental tasks including establishing intimate relationships. In contrast, those who have not yet succeeded in this developmental task of young adulthood, appear to be at risk for lower life satisfaction.

People in committed romantic relationships reported less romantic loneliness than single individuals, as predicted by H2. These findings support the notion that the lack of romantic partners or intimate relationships may be an important causal factor for one’s present feelings of loneliness (Rokach and Brock 1998). This finding replicates others in the literature showing that involvement in a romantic relationship is significantly associated with lower levels of romantic loneliness (DiTommaso and Spinner 1993).

Contrary to our expectation, partnered and single individuals did not differ in regard to dating anxiety. The current study suggests that single individuals may not manifest lower level of dating anxiety, compared to their partnered individuals. This sample size of this study generated statistical power of .80 to detect an effect as small as *d* = .30, so low statistical power can be ruled out as a cause of this null finding. One possibility is that single participants had that relationship status by choice, not because they were too anxious to establish dating relationships. Second, with the advent of modern communication technologies and social media, there are many more low threat mechanisms for relationship formation that might weaken the observed association between dating anxiety and involvement in a romantic relationship. Also, as Eddington et al. (2010) suggested, the Dating Anxiety Scale for Adolescents (DAS-A) exclusively measures cognitive aspects of dating. To be precise, this measure may not necessarily address affective components of dating activity (Eddington et al. 2010).

Based on prior research we expected that young adults in committed relationships would exhibit greater interpersonal competence. This prediction was not supported by the findings from this study. Young adults in romantic relationships were assumed to possess greater social competence, reflecting their ability to engage successfully in social interactions and interpersonal relationships (Larson et al. 2007). It is at least possible that success in romantic relationships requires some different interpersonal competencies than those measured by the Interpersonal Competence Questionnaire-Revised (ICQ-R; Buhrmester et al. 1988). For example, warmth, trust, and reciprocity play an important role in young adult romantic relationships (Donnellan et al. 2005; Larson et al. 2007). Also, just as with dating anxiety, it is possible that single participants might not be in that relational state due to a trait like deficiency (e.g., poor social skills, high anxiety) but rather, by their own choice. In addition, the findings from this investigation indicate that single individuals report levels of social competence that are equivalent to those of their peers who are in romantic relationships. This suggests that romantic relationship status may not be the best reflection of social competencies, and that single individuals may effectively cultivate social competences in other relational contexts (e.g., family, platonic friendship, work).

The structural model that tested H5 and H6 yields some insights into how and why interpersonal competencies are associate with romantic desolation and why dating anxiety may degrade life satisfaction, namely through several indirect effects. For example, interpersonal competence was strongly associated with lower dating anxiety, and lower dating anxiety was associated with lower romantic desolation (i.e., being single and having high romantic loneliness). This indirect effect shows that ease in social settings such as dating, might be one of the benefits of possessing interpersonal competence and this may contribute to greater propensity to experience romantic fulfillment during young adulthood. Second, there was an indirect effect of dating anxiety on life satisfaction, through romantic desolation. This effect explains why dating anxiety may be deleterious to life satisfaction: Because people with this type of anxiety may find it difficult to form and sustain romantic relationships and because they are also at risk for experiencing high levels of romantic loneliness. Documentation of these two significant indirect effects reveals plausible answers as to why low interpersonal competence is a risk for romantic desolation and why dating anxiety creates a comparable risk for low life satisfaction during young adulthood.

## Limitations and Future Directions

There are several limitations of this study. First, the correlational nature of the study precludes any causal inferences concerning the associations between interpersonal competence, romantic involvement, dating anxiety, and life satisfaction. In addition, we have presented preliminary evidence for a model of interpersonal competence and life satisfaction, mediated by dating anxiety and romantic desolation. At this point, it would be useful to test this model prospectively with longitudinal data. Second, all participants were never married, heterosexual, childless, and residing in a large city. In addition, the age of the sample, even though representing a unique developmental state, precludes any generalizations to individuals in middle and late adulthood. In future studies it would be useful to compare young adults with the adjustment of single and partnered individuals in middle and late adulthood. Third, in the current study we did not measure the quality of the relationships among partnered individuals. In future research it would be useful to include the measure of the quality of the relationships as prior research shown, for example, that marital quality may act as an intensifier of health and well-being (DePaulo and Morris 2005). In addition, in subsequent research it would be fruitful to include the measure of affective aspects of dating anxiety as the Dating Anxiety Scale used in the current study exclusively measures cognitive aspects of dating. Fourth, it would also be useful to assess how long participants remained single or how long they were involved in a relationship, as well as whether they were voluntarily single and not seeking out a romantic partner. Each of these variables could exhibit unique associations with constructs such as loneliness, dating anxiety, interpersonal competence, and life satisfaction. This recommendation is supported by a recent study (author citation) demonstrating, for instance that individuals who perceive their singlehood as voluntary report lower romantic loneliness than individuals who perceive their singlehood as involuntary.

It is important to note that interpersonal competence, romantic involvement, and dating anxiety do not fully explain the life satisfaction of young adults. At the same time, it is clear that interpersonal competence, dating anxiety, and romantic desolation are substantial elements in a comprehensive explanation of successful developmental task achievement and life satisfaction in young adulthood. In our opinion, understanding the interplay between dating anxiety and the more developmental constructs such as competence (in particualar, heterosocial competence) and romantic involvment will provide a more compehensive picture of the determinants of life satisfaction in young adulthood.

Despite the enumerated limitations of the study, the present findings underscore the importance of interpersonal competence as young adults take on the developmental task of cultivating romantic attachments. Interpersonal competence appears to be positively associated with higher life satisfaction through lower dating anxiety and low dating anxiety is positively associated with life satisfaction through lower romantic desolation.
